# Evaluating Adverse Drug Reactions, Their Reporting Rates and Their Impact on Attitudes Toward Pharmacotherapy Among Female Patients with Schizophrenia: Insights and Implications from a Cross-Sectional Study

**DOI:** 10.3390/healthcare12242595

**Published:** 2024-12-23

**Authors:** Josipa Bukić, Dora Herceg, Darko Modun, Ivana Krce, Dario Leskur, Toni Durdov, Miroslav Herceg, Ana Šešelja Perišin, Doris Rušić

**Affiliations:** 1 Department of Pharmacy, University of Split School of Medicine, Soltanska 2A, 21000 Split, Croatia; josipa.bukic@mefst.hr (J.B.); darko.modun@mefst.hr (D.M.); ivana.krce142@gmail.com (I.K.); toni.durdov@mefst.hr (T.D.); aperisin@mefst.hr (A.Š.P.); doris.rusic@mefst.hr (D.R.); 2 Department of Laboratory Medicine and Pharmacy, Faculty of Medicine, Josip Juraj Strossmayer University of Osijek, Josipa Huttlera 4, 31000 Osijek, Croatia; 3 School of Medicine, University of Zagreb, Šalata 3, 10000 Zagreb, Croatia; doherceg@gmail.com (D.H.); miroslav.herceg@bolnica-vrapce.hr (M.H.); 4 University Psychiatric Hospital Vrapče, Bolnička cesta 32, 10090 Zagreb, Croatia

**Keywords:** schizophrenia, female patients, attitudes, adherence, adverse drug reactions

## Abstract

Background/Objectives: Schizophrenia is a chronic psychiatric disorder usually managed with antipsychotics, which can cause adverse drug reactions (ADRs) that may impact patients’ attitudes toward their treatment, as well as treatment adherence. This study aimed to assess the influence of ADRs and other factors on treatment attitudes among female patients with schizophrenia. Methods: A cross-sectional study was conducted at the Vrapče Psychiatry Clinic with 109 female schizophrenia patients. The DAI-10 (Drug Attitude Inventory) questionnaire was used to assess attitudes toward treatment. Data on their demographic details, pharmacotherapy, ADR occurrence and ADR reporting rates were collected. Multiple regression analyses were used to identify predictors of DAI-10 scores. Results: Patients using more medications and those experiencing ADRs had lower DAI-10 scores, indicating less favorable attitudes (F (2, 106) = 7.364, *p* = 0.001, R^2^ = 0.105). ADRs, primarily extrapyramidal symptoms and weight gain, were reported by 43.1% of patients; however, only one patient formally reported them. First-generation antipsychotics were associated with a higher prevalence of ADRs (χ^2^ = 4.969, df = 1, *p* = 0.022). Conclusion: Negative experiences with ADRs significantly impact patients’ attitudes and adherence. Low ADR reporting rates highlight the need for better pharmacovigilance education. Enhancing patient awareness may foster more positive attitudes and adherence, potentially improving patient outcomes.

## 1. Introduction

Schizophrenia is a severe psychiatric disorder with a chronic and limiting course characterized by the occurrence of psychoses [1]. The etiology of schizophrenia is not fully elucidated; however, research indicates various abnormalities in brain structures and brain function [2,3]. Inflammation is also implicated in the etiology of schizophrenia, with peripheral markers of inflammation potentially serving as diagnostic indicators [4]. The clinical image of schizophrenia is characterized by positive and negative symptoms and impairment of a patient’s cognitive function. Positive symptoms of schizophrenia attract the most attention and are best controlled with pharmacotherapy [5,6]. The treatment of schizophrenia is currently based on the use of antipsychotics, and this use is associated with specific adverse drug reactions (ADRs). First-generation antipsychotics cause pronounced movement disorders, such as parkinsonism, tardive dyskinesia, dystonia and akathisia [7]. The second group of antipsychotics, called atypical antipsychotics, can cause metabolic adverse drug reactions and increase the risk of weight gain. One study also found that lower HDL cholesterol levels were associated with increased aggression in schizophrenic patients, meaning that metabolic health may influence not only physical but also behavioral outcomes in schizophrenia [8]. However, studies show a positive impact of metformin in combination with second-generation antipsychotics on the reduction in metabolic effects [9,10,11].

Research shows that patients with schizophrenia have a greatly reduced quality of life and life expectancy, with age, the number of psychoses and hospitalizations, the length of the period without treatment and ADRs of antipsychotics affecting their life. In addition, the high rate of unemployment among people with schizophrenia also threatens their quality of life. To combat this, studies have shown that regular check-ups by a psychiatrist and good adherence to therapy could improve their quality of life [12,13]. Attitudes of schizophrenic patients toward their medication regiment were also proven as highly important factors for patients’ adherence to their treatment [14]. Adverse drug reactions could significantly lower patients’ opinion on the drugs they are using and consequently lower adherence levels [15,16]. The Drug Attitude Inventory (DAI) is a long-standing questionnaire developed in 1983, and it is still the most commonly used tool for assessing attitudes toward drugs among patients with mental illness. DAI-10 is the shorter, 10-question variation of the DAI, with DAI-30 also used in practice [17]. A previous study identified differences between female and male schizophrenia patients in the factors causing negative attitudes toward their medication, with different psychological, social and clinical factors contributing toward negative attitudes in females compared to males [18]. Further, the incidence, clinical characteristics and progression of disease, the response to therapy and outcomes vary between genders [18,19,20,21,22]. This disparity between genders warrants further investigation. It would be beneficial to identify factors associated with poorer attitudes among the female population in a Croatian outpatient setting to allow for more appropriate treatment of this patient group in the future based on their specific needs and characteristics [18].

The main goal of this study was to examine the effects of ADRs and other relevant factors on schizophrenic patients’ attitudes toward their prescribed medication using the DAI-10 questionnaire [23]. The secondary goal was to analyze adverse drug reactions reported by patients and patients’ tendency to report them to healthcare professionals or authority. To our knowledge, this is the first study focusing on factors influencing attitudes toward antipsychotic medication in Croatian female patients with schizophrenia. Furthermore, the study investigates patients’ reporting behaviors regarding ADRs, an often neglected but important aspect of the public health system required for accurate determination of the safety of medication.

## 2. Materials and Methods

The research was approved by the Ethics Committee of the Vrapče Psychiatry Clinic and was organized as a cross-sectional study. Respondents included in the study were surveyed on the premises of the Institute for Female Psychotic Disorders of the Vrapče Psychiatry Clinic in the period from May to October 2022. The inclusion criteria for respondents were female gender, outpatient treatment, legal age and a confirmed diagnosis of schizophrenia. A diagnosis was made in accordance with the Diagnostic and Statistical Manual of Mental Disorders (DSM-5) criteria [5]. Continuous symptoms (for at least six months) had to be present to be included in the study. Patients with schizoaffective disorder and depressive or bipolar disorder with psychotic features were not included. Patients with secondary psychoses were also not included. Further exclusion criteria included being hospitalized in the previous year, inadequate social functioning and insufficient work capability [23].

The research was preceded by a search of the available scientific literature to select a suitable questionnaire for patients with schizophrenia. Due to its appropriateness in the mentioned population, the DAI scale was chosen to assess drug satisfaction in study participants. The scale was translated from English to Croatian before conducting the research [23]. This tool contains only ten questions (statements): six of them represent positive attitudes toward antipsychotic treatment and four have negative connotation. The patient marks each statement as true or false according to whether the statement applies to them. Answering “True” on the positive statement awarded 1 point and answering “False” awarded −1 point, while answering “True” on the negative statement awarded −1 point and answering “False” awarded 1 point. The possible total score was from −10 to 10 points. A higher number of points indicates a more positive attitude toward antipsychotic treatment and is associated with better adherence in patients [17,24].

The final version of the survey consisted of additional questions which determined the demographic data of the respondents and questions about pharmacotherapy. Data were collected on the age, marital status and employment status of the patients. Furthermore, data were collected on the drug used (generic name and dosage), method of use (oral or intramuscular), duration of drug use, and ADRs of the drugs used and whether they were reported to the relevant authority [23].

A chi-square test was conducted to compare adverse drug reaction presence based on the medications used by the patients. Logistic regression was used to assess the influence of the number of drugs in the therapy regiment on adverse drug reaction presence. Multiple regression was performed to determine what predictors influence the total DAI-10 score of the patient. Multiple regression analysis was chosen because it enabled quantification of predictors’ influence on the outcome (DAI-10 score). Assumptions for multiple regression analysis, including linearity, homoscedasticity and normality of residuals, were verified to ensure the validity of the model. A stepwise method was used to find the most accurate model of DAI-10 score prediction. The dependent variable was the DAI-10 score and the independent variables were as follows: age, the number of used medications, the duration of medication usage, medication route of administration (oral or intramuscular), the presence of first-generation antipsychotics in the therapy regimen and the presence of adverse drug reactions. Predictors were chosen based on previous studies on the influence of different factors on treatment adherence and patients’ perceptions of their own pharmacotherapy. The predictors were all tested for their individual effect on DAI-10 score using single-predictor linear regression. All statistical analyses were performed with the IBM SPSS Statistics software (version 25). Statistical significance was set at 0.05.

The methodology is further detailed in Supplementary File S1, where the steps are outlined in pseudocode for additional clarity. More details about each step of the regression analysis is provided in Supplementary File S2.

## 3. Results

### 3.1. Demographic Characteristics

A total of 109 patients who visited the Institute for Female Psychotic Disorders of the Vrapče Psychiatry Clinic during the period under investigation participated in the study and gave their informed consent to participate in the study. The demographic characteristics of the respondents are shown in Table 1. The majority of the respondents were between 50 and 60 years old, did not have a partner and were not employed.

### 3.2. Pharmacotherapy

The antipsychotics used in the examined patients and the frequency of their use are shown in Table 2. A total of 88 patients (80.7%) used only one drug in their therapy regimen, while 21 patients (19.3%) used two to four antipsychotics simultaneously. The most frequently used drug was olanzapine, in 15.5% of patients, followed by paliperidone and clozapine. Almost a third of patients, 31.2%, used drugs from the first generation of antipsychotics (haloperidol, promazine, fluphenazine and sulpiride). In total, 72.5% of patients used an orally administered drug, and the rest used an intramuscularly administered antipsychotic.

In addition to antipsychotics, the respondents also used medications from other pharmacotherapeutic groups. Only 11% of respondents used one medication, 45.9% used two, 22.9% used three and 20.2% of respondents used four or more medications.

### 3.3. Adverse Drug Reactions

A total of 47 respondents, which is 43.1%, stated that they had experienced an adverse drug reaction to the antipsychotic used. Out of the 47 who had experienced ADRs, only 1 (2.1%) had reported them to the healthcare professional. Low ADR reporting rates could be a sign of obstacles in communication between the patients and the prescribers, patients’ lack of motivation to report ADRs and a lack of knowledge about the importance of reporting ADRs.

The types of ADRs reported by patients are shown in Table 3. The most frequently recorded ADRs were extrapyramidal syndromes and weight gain, both in 28.3% of the subjects.

Patients who had first-generation antipsychotics in their therapy regimen more often experienced ADRs compared to those who did not use the aforementioned drugs (58.8% vs. 41.2%, using first-generation antipsychotics and not using first-generation antipsychotics; χ^2^ = 4.969; df = 1, *p* = 0.022).

### 3.4. Factors Associated with DAI-10

**Figure 1:**
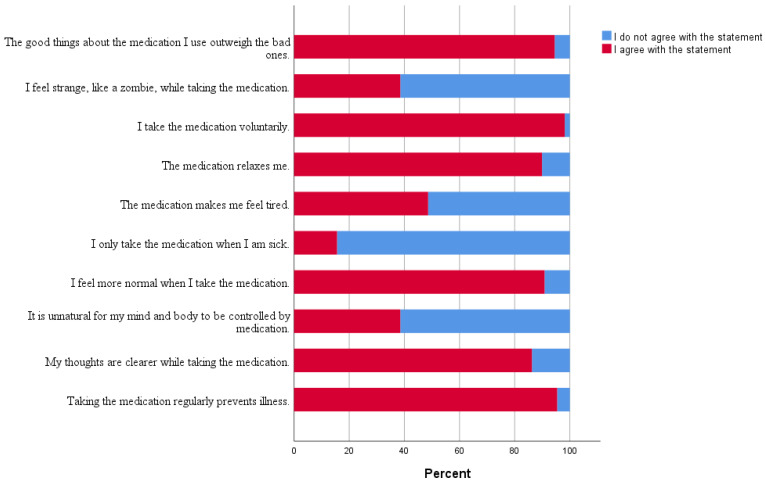
The percentage of answers given by participants on individual DAI-10 statements.

As Figure 1 depicts, the vast majority of participants considered that the good things about the medication outweigh the bad ones. Around half of the participants blamed the drugs for feeling tired.

Prior to assigning predictors to the stepwise multiple linear regression, they were analyzed as single predictors of DAI-10 score to assess their individual contribution. As seen in Table 4, the number of used medication (R^2^ = 0.087) and adverse drug reaction presence (R^2^ = 0.044) were the predictors with the highest percentage of variation in DAI-10 score.

Step two of the multiple regression found the most accurate model. Independent variables significantly influence DAI-10 score, (F (2, 106) = 7.364, *p* = 0.001), which indicates that the selected model had an impact on DAI-10 score. However, the adjusted R^2^ = 0.105 shows that the model explains only 10.5% of the variance in DAI-10 score. There are other factors, not included in this study, which influence DAI-10 score. When assessing the individual impact of the selected factors on DAI-10 score, the number of used medications significantly influences DAI-10 score (β = −0.280, *p* = 0.003), as seen in Table 5. Patients who took more than one medication had on average a DAI-10 score 0.64 points lower, adjusted for all other predictors. Also, patients who experienced ADRs on average had a DAI-10 score of 6.89 points (95% CI 5.70–7.70) compared to a score of 8.13 points (95% CI 6.83–9.72). Any adverse drug reaction significantly impacts DAI-10 score (β = −0.187, *p* = 0.0439).

## 4. Discussion

The results of our study indicate that attitudes toward the use of antipsychotic drugs are associated with the number of drugs which patients use and the presence of ADRs to antipsychotic drugs.

The results of this study also indicate positive attitudes toward the use of antipsychotics among female schizophrenic patients, even among those who expressed concerns about potential ADRs of the medication. The majority of patients reported feeling clearer-headed while on the medication and believed that regular use helped prevent the recurrence of their illness. Notably, 98% of all patients voluntarily adhered to their medication, which positively influenced both their adherence and their attitudes toward schizophrenia pharmacotherapy. Similar findings were observed in a study by Hatano et al., which showed that patients who chose their pharmacotherapy in collaboration with their physician exhibited more positive attitudes toward antipsychotic drug use than those whose treatment was solely physician-directed [24].

This study showed that only one patient reported a suspected adverse reaction of antipsychotics. This is a disappointing result, especially considering that in Croatia, patients themselves can report suspected ADRs, which is not yet possible in many countries. In Croatia, suspected ADRs can also be reported via a mobile application. Croatia was among the first EU countries to implement ADR reporting through a mobile app, and preliminary research has shown an increase in reports through this channel [25]. Interestingly, previous studies have shown that women tend to report suspected ADRs more frequently than men, which would suggest a higher proportion of reports in this female patient population. For this reason, there is a need to raise awareness of the possibility and importance of reporting suspected ADRs among patients taking antipsychotics and to increase knowledge about their safety and the frequency of specific types of ADRs [26].

Interestingly, most patients in this study belonged to the age group between 50 and 60 years. This finding may be explained by previous research indicating a second peak age for the onset of schizophrenia symptoms in women. While the highest incidence in men occurs during adolescence, women may experience a second “peak” in their fifties, likely due to hormonal changes. However, these claims require further epidemiological studies and a more detailed analysis of the mechanisms underlying this phenomenon in postmenopausal women [2].

Investigating predictors of patients’ non-adherence was the goal of a few recent studies [27]. The results of a study by Ngui and colleagues involving 8595 participants point to possible factors affecting antipsychotic adherence. One identified factor is the use of second-generation antipsychotics, which was statistically significantly associated with better adherence in both male and female patients compared to the use of first-generation antipsychotics. Another observed factor is the patient’s age, with results showing that older patients tend to be more adherent compared to younger schizophrenia patients. Gender also affects adherence, as 13.71% of female patients in the study were adherent, compared to 11.01% of male patients. This difference may be due to sexual dysfunction caused by antipsychotics, which has a greater impact on men than on women [28]. A recent study conducted in the US confirmed younger age as a poor adherence factor in addition to previous hospitalization and more frequent dosing of the medication [29].

Poor adherence to antipsychotic therapy is one of the common reasons for relapse in schizophrenia and the hospitalization of patients, which not only contributes to poor schizophrenia control but also increases healthcare system costs [30]. One way to improve adherence to antipsychotic medication is choosing a drug that the patient will tolerate well. Additionally, there is an emphasis on the need to educate patients about the importance of regular pharmacotherapy use [31]. Studies have shown that only half of schizophrenia patients comply with the medication regiment, as expected [32].

This study, though among the first in Croatia and the European Union to examine the impact of ADRs on schizophrenia pharmacotherapy exclusively in female patients, has certain limitations. Firstly, the exclusion of male participants limits the ability to draw direct comparisons between genders. Additionally, the regression analysis did not take into account clinical and social aspects that might have influenced adherence. Previous studies have shown that patients’ insights about mental disorders and their therapy, other clinical aspects such as the number of relapses and illness severity, patients’ socioeconomic status and social support, and health system barriers could greatly affect therapy adherence [33]. As our study did not collect data on these variables, the ability to fully explore their potential impact on the findings was limited. Moreover, the sample size was relatively small, although we believe the focus on female participants still provides valuable insights for future research in this population. Therefore, future studies should include equivalent male controls and larger, more diverse samples, which should enable us to better understand the interactions between clinical, social and other variables with therapy adherence. Another limitation is that it was a single-center study, as it was only conducted at the Department for Female Psychotic Disorders at the Vrapče Psychiatric Clinic in Zagreb. Future research should include departments from other institutions, ideally in various cities. As with any survey-based study, recall bias and the potential for socially desirable responses may affect the data. Moreover, the observed low number of ADRs among participants may relate to the length of treatment, as some ADRs may diminish or disappear over time, or patients may become accustomed to them and no longer perceive them as ADRs worth reporting. This study was conducted using the DAI-10 questionnaire; however, other tools exist that were used in other studies to assess patients’ attitudes. One of them is DAI-30, an original and longer version of the DAI. Similar studies used other questionnaires developed specifically for antipsychotic medication, for example the Rating of medication influences (ROMI) scale [34,35], the Subjective Well-being under Neuroleptic Treatment Scale (SWN) [36] or self-made questionnaires such as the Attitude and Status toward Treatment of Community Patients with Schizophrenia Questionnaire (AST-CSQ) [37]. General questionnaires not purposely built for the assessment of antipsychotics, such as the Belief about Medicines Questionnaire, were used in some studies [38]. The results of studies using different tools to measure attitudes are not completely comparable and must be analyzed cautiously. However, most studies used a variant of the DAI. The DAI-10 used in this study is a shorter version, but it is still highly correlated with the longer and more comprehensive DAI-30, while being much simpler for patients to fill out [17,39]. Despite these limitations, the results of this study point to a significantly low awareness of the importance of reporting ADRs for this class of drugs and highlight the need for education on the basics of pharmacovigilance, particularly among patients with schizophrenia.

## 5. Conclusions

To conclude, adverse drug reactions and the complexity of medication regimens were important factors impacting attitudes toward antipsychotic medications in female patients with schizophrenia. Patients with more medications included in their therapy regimen and those experiencing ADRs had less favorable attitudes which might influence their therapy adherence. First-generation antipsychotics were associated with a higher prevalence of ADRs. This study also found a low rate of ADR reporting, highlighting the need for increased awareness and education on pharmacovigilance among patients. More research is needed to confirm these finding, as well as to explore additional factors that might influence patients’ attitudes and adherence [23].

## Figures and Tables

**Table 1:** Demographic characteristics of respondents.

Characteristic	N (%)
Age	
20–29	5 (4.6)
30–39	15 (13.8)
40–49	30 (27.5)
50–59	44 (40.4)
>60	15 (12.7)
Relationship status	
Single	37 (33.9)
In a relationship	8 (7.3)
Married	29 (26.6)
Divorced	22 (20.2)
Widow	13 (11.9)
Employment status	
Unemployed	60 (55.0)
Employed	49 (45.0)

**Table 2:** Antipsychotics and frequency of use in test subjects.

Drug	N (%)
Phenothiazines	
Promazine	14 (10.4)
Fluphenazine	8 (5.9)
Butyrophenones	
Haloperidol	14 (10.4)
Benzamides	
Sulpiride	4 (2.9)
Tricyclics	
Clozapine	16 (11.8)
Quetiapine	12 (8.8)
Olanzapine	21 (15.5)
Benzisoxazoles	
Risperidone	13 (9.6)
Paliperidone	18 (13.3)
Phenylpiperazines	
Aripiprazole	15 (11.1)
Total	135 (100)

**Table 3:** Adverse drug reactions in research subjects.

Adverse Drug Reactions	N (%)
Decreased libido	2 (3.7)
Extrapyramidal symptoms (tremor, dystonia)	15 (28.3)
Hypersalivation	6 (11.3)
Amenorrhea	1 (1.9)
Increase in body weight	15 (28.3)
Sedation	7 (13.2)
Headache	2 (3.7)
Instability	1 (1.9)
Hyperprolactinemia	1 (1.9)
Lack of concentration	1 (1.9)
Constipation	1 (1.9)
Depression	1 (1.9)
Total:	53

**Table 4:** Single-predictor liner regression of selected predictors later used in multiple linear regression.

Independent Variable	B [95% CI]	β	*p*-Value	R^2^
Age	0.271 [0.091; 0.452]	0.089	0.003	0.008
Number of Used Medications	−0.675 [−0.804; −0.0545]	−0.295	<0.001	0.087
Adverse Drug Reaction Presence	−1.382 [−1.765; −0.988]	−0.209	<0.001	0.044
Duration of Medication Usage	0.006 [0.000; 0.000]	0.064	0.035	0.004
Medication Route of Administration	0.488 [0.014; 0.882]	0.061	0.043	0.004
Presence of First-Generation Antipsychotics	−1.340 [−1.752; −0.928]	−0.190	<0.001	0.036

**Table 5:** Multiple linear regression analysis for factors associated with DAI-10 score (step 2).

Variable	B [95% CI]	β	*p*-Value	R^2^	ΔR^2^
Number of used medications	−0.641 [−1.093; −0.256]	−0.295	0.002	0.122	0.035
Adverse drug reaction presence *	−1.236 [−2.430; −0.042]	−0.187	0.0043		

CI—confidence interval. Excluded: age, duration of medication usage, medication route of administration and presence of first-generation antipsychotics in therapy regimen. * Reference group was patients who had not experienced adverse drug reaction.

## Data Availability

The data are available upon reasonable request to the corresponding author.

## Supplementary Materials

The following supporting information can be downloaded at: https://www.mdpi.com/article/10.3390/healthcare12242595/s1: Supplementary File S1: Pseudocode illustrating the research methodology; Supplementary File S2: Regression—additional data.
