# Supplementary material for: Evaluating Adverse Drug Reactions, Their Reporting Rates and Their Impact on Attitudes Toward Pharmacotherapy Among Female Patients with Schizophrenia: Insights and Implications from a Cross-Sectional Study
Source: Healthcare (Basel). 2024 Dec 23;12(24):2595. doi: 10.3390/healthcare12242595 (PMC11727682; doi:10.3390/healthcare12242595)
Supplement: Supplementary file 1 [file healthcare-12-02595-s001.zip › Supplementary File S1 Pseudocode.pdf]

Supplementary File S1: Pseudocode illustrating the research methodology

```
BEGIN

  // Ethics Approval and Study Setup
  APPROVE study by Ethics Committee of Vrapče Psychiatry Clinic
  DEFINE study type as Cross-sectional study
  SET study period from May to October 2022
  SET study location at Vrapče Psychiatry Clinic, Institute for Female Psychotic Disorders

  // Define inclusion criteria
  INCLUDE participants who meet the following:
    Female gender
    Outpatient treatment status
    Legal age
    Confirmed diagnosis of schizophrenia based on DSM-5 criteria
    Continuous symptoms for at least six months
  EXCLUDE participants who meet any of the following:
    Schizoaffective disorder
    Depressive or bipolar disorder with psychotic features
    Secondary psychoses
    Hospitalized in the previous year
    Inadequate social functioning
    Insufficient work capability

  // Literature Review and Questionnaire Selection
  SEARCH scientific literature for a suitable questionnaire
  SELECT DAI scale (Drug Attitude Inventory) for assessing drug satisfaction
  TRANSLATE DAI scale from English to Croatian
```

```
// The DAI scale consists of 10 questions: 6 positive, 4 negative

// Administering the Questionnaire
FOR each participant DO:
    PRESENT the DAI scale questions to participant
    COLLECT answers (True/False)
    CALCULATE score:
        // Positive statements: "True" = +1, "False" = -1
        // Negative statements: "True" = -1, "False" = +1
    SUM total score (from -10 to 10)

// Collect Demographic and Pharmacotherapy Data
FOR each participant DO:
    COLLECT demographic data (age, marital status, employment)
    COLLECT pharmacotherapy data (drug name, dosage, route of administration, duration of use)
    COLLECT ADR (Adverse Drug Reaction) information and whether ADR was reported

// Statistical Analysis
// Chi-Square Test: Compare ADRs based on medication used
FOR each medication group DO:
    CONDUCT Chi-square test to compare ADR presence based on medication used

// Logistic Regression: Assess the influence of number of drugs on ADRs
CONDUCT logistic regression to assess impact of number of drugs on ADR presence

// Multiple Regression: Determine predictors influencing DAI-10 score
CONDUCT multiple regression analysis to identify predictors of DAI-10 score:
    DEPENDENT variable: DAI-10 score
```

INDEPENDENT variables: age, number of drugs, duration of use, medication route, first-generation antipsychotics presence, ADR presence

// Step-wise Regression: Find the most accurate model for DAI-10 score prediction

PERFORM stepwise method for regression to identify best predictors for DAI-10 score

// Statistical significance

SET significance level at 0.05

PERFORM all statistical analysis using IBM SPSS Statistics (version 25)

END
