# Supplementary material for: Evaluating Adverse Drug Reactions, Their Reporting Rates and Their Impact on Attitudes Toward Pharmacotherapy Among Female Patients with Schizophrenia: Insights and Implications from a Cross-Sectional Study
Source: Healthcare (Basel). 2024 Dec 23;12(24):2595. doi: 10.3390/healthcare12242595 (PMC11727682; doi:10.3390/healthcare12242595)
Supplement: Supplementary file 1 [file healthcare-12-02595-s001.zip › Supplementary File S2 Regression - additional data.pdf]

Supplementary Table S1. Coefficient of determination values for each step of regression

Model	R	R^2	Adjusted R^2	ΔR^2
1. Number of used Medications	0.295	0.087	0.079	0.087
2. + Adverse Drug Reaction Present	0.349	0.122	0.105	0.035
3. +Age	0.374	0.140	0.116	0.018
4. +Medication Route of Administration	0.385	0.148	0.115	0.008
5. +Presence of First – Generation Antipsychotics	0.386	0.149	0.108	0.001
6. +Duration of Medication Usage	0.386	0.149	0.099	0.000
